# Sex Differences in Combined Effects of Maternal Pre‐Pregnancy BMI and OGTT Response Patterns on Early Neurodevelopment in Offspring: A Cohort Study

**DOI:** 10.1111/1753-0407.70261

**Published:** 2026-08-11

**Authors:** Kai Chen, Xiaoxuan Fan, Yuji Wang, Zhongqiang Cao, Luli Xu, Xiya Qin, Mingzhao Huang, Xiaofeng Mu, Chao Xiong, Aifen Zhou

**Affiliations:** ^1^ Data Center, Wuhan Children's Hospital (Wuhan Maternal and Child Healthcare Hospital), Tongji Medical College, Huazhong University of Science and Technology Wuhan China; ^2^ Department of Medical Records Statistics Wuhan Children's Hospital (Wuhan Maternal and Child Healthcare Hospital), Tongji Medical College, Huazhong University of Science and Technology Wuhan China; ^3^ Institute of Maternal and Child Health, Wuhan Children's Hospital (Wuhan Maternal and Child Healthcare Hospital), Tongji Medical College, Huazhong University of Science and Technology Wuhan China

**Keywords:** cohort study, gestational hyperglycemia, neurodevelopment, oral glucose tolerance test (OGTT), pre‐pregnancy obesity

## Abstract

**Introduction:**

Maternal pre‐pregnancy overweight/obesity and gestational hyperglycemia are linked to adverse offspring neurodevelopment, but the role of oral glucose tolerance test (OGTT) response patterns remains unclear. We investigated the independent and combined effects of maternal pre‐pregnancy BMI and OGTT response patterns on neurodevelopment in 2‐year‐old offspring.

**Methods:**

This cohort study included 2639 mother–child pairs from Wuhan, China. A 75‐g OGTT was performed at 24–28 gestational weeks, with glucose measured at fasting, 1 and 2 h. Group‐based trajectory modeling identified distinct OGTT response patterns. Offspring neurodevelopment was assessed at 2 years using the Bayley Scales of Infant Development. Associations were examined with generalized linear and logistic regression models, and additive interaction was evaluated using RERI and AP.

**Results:**

Three OGTT response patterns were identified; pattern 3 showed the most pronounced glycemic response. Maternal pre‐pregnancy overweight/obesity alone showed nominal associations with lower MDI scores in the overall population and in girls, but these associations did not remain significant after FDR correction. In girls exposed to both maternal overweight/obesity and pattern 3, MDI was markedly lower (*β* = −13.73, 95% CI: −23.06 to −4.39), and the risk of suboptimal mental development was higher (OR = 2.88, 95% CI: 1.03 to 8.09), with significant additive interaction (RERI = 0.76; AP = 0.42).

**Conclusions:**

Maternal pre‐pregnancy overweight/obesity and OGTT response patterns alone showed limited independent associations with offspring neurodevelopment. However, combined exposure may be associated with poorer mental developmental performance, with more consistent evidence in girls.

## Highlights

- Routine three‐point OGTT data identified distinct maternal glucose response patterns in a Chinese birth cohort.
- Maternal pre‐pregnancy BMI and OGTT response patterns showed limited independent associations with offspring neurodevelopment after FDR correction.
- Combined exposure to pre‐pregnancy overweight/obesity and a pronounced OGTT response pattern was associated with poorer mental developmental performance.
- Evidence for the adverse combined effect was more consistent in girls, supported by both continuous MDI scores and suboptimal mental development.

## Graphical abstract

Maternal pre‐pregnancy overweight/obesity, when combined with a pronounced OGTT glucose‐response pattern, was associated with poorer mental development in 2‐year‐old daughters, highlighting the importance of preconception weight management and glycemic control during pregnancy.

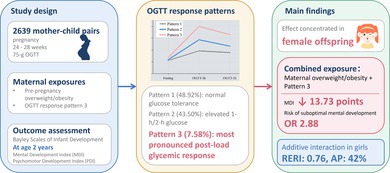

**Abbreviations**
AP: attributable proportion due to interaction
BIC: bayesian information criterion
BMI: body mass index
BSID: bayley scales of infant development
CI: confidence interval
FDR: false discovery rate
GBTM: group‐based trajectory modeling
GDM: gestational diabetes mellitus
IOM: Institute of Medicine
MDI: mental development index
OGTT: oral glucose tolerance test
OR: odds ratio
PDI: psychomotor development index
RERI: relative excess risk due to interaction
SD: standard deviation

## 1 Introduction

Maternal obesity is a prevalent condition that adversely affects offspring health [1]. Pre‐pregnancy or gestational overweight/obesity correlates with neurodevelopmental impairments in children, including cognitive, behavioral and emotional problems [2, 3, 4, 5]. Obesity is closely linked to chronic inflammation, a key contributor to insulin resistance [6, 7]. Compared to women with normal weight, those with pre‐pregnancy obesity exhibit more severe insulin resistance, which exacerbates dysregulation of glucose metabolism and compromises glycemic control during pregnancy [1, 7]. Furthermore, maternal obesity combined with inadequate glycemic control can impair placental metabolic and endocrine function. This predisposes the fetus to the detrimental effects from maternal chronic inflammation [8], hyperglycemia [9], and insulin resistance [7], ultimately leading to long‐term adverse outcomes in fetal organ development and function.

Emerging epidemiological evidence has also established associations between suboptimal glycemic control during gestation and adverse neurodevelopmental outcomes in offspring [10, 11]. However, most studies on maternal metabolic status have focused primarily on the diagnosis of gestational diabetes mellitus (GDM). For example, a systematic review of 19 cohorts suggested that children exposed to maternal GDM have an elevated risk of developing autism spectrum disorders and attention‐deficit/hyperactivity disorders [10]. A Finnish registry‐based study of approximately 400 000 individuals indicated that maternal GDM was associated with offspring being diagnosed with neurodevelopmental or mental disorders before 11 years of age [11]. In China, GDM screening is typically performed using a 75‐g oral glucose tolerance test (OGTT) at 24–28 weeks of gestation, with diagnosis based on whether any of the fasting, 1‐ or 2‐h blood glucose levels exceed established cutoff values [12]. Notably, the shape of the glycemic response curve across multiple OGTT time points provides valuable insight into pancreatic *β*‐cell function [13, 14, 15]. Nevertheless, previous studies [10, 11] dichotomize gestational glycemia using GDM diagnosis, potentially overlooking informative heterogeneity in the OGTT glucose response curve.

Therefore, utilizing data from a birth cohort in Wuhan, China, this prospective study aimed to characterize OGTT response patterns and examined their independent and joint associations with offspring neurodevelopment.

## 2 Methods

### 2.1 Study Design and Population

This study utilized data from a birth cohort established at Wuhan Children's Hospital (Wuhan Maternal and Child Health Hospital). The cohort included pregnant women who registered for prenatal care and delivered at the hospital between May 2014 and May 2019. All participants underwent a 75‐g oral glucose tolerance test (OGTT) at 24–28 weeks of gestation, and their children were assessed for neuropsychological development using the Bayley Scales of Infant Development (BSID) at 2 years of age.

**FIGURE 1:**
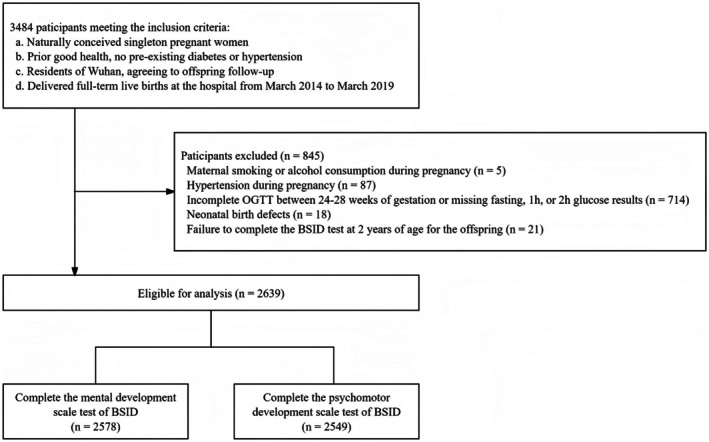
Flowchart of study participant inclusion and exclusion.

A total of 3484 mother–child pairs met the inclusion criteria. Of these, 2639 were included in the final statistical analysis, comprising 2578 offspring who completed the mental development scale and 2549 who completed the psychomotor scale. Figure 1 illustrates the detailed inclusion and exclusion criteria along with the participant flow diagram.

This study was approved by the Ethics Committee of Wuhan Children's Hospital (Wuhan Maternal and Child Health Hospital) (approval number: 2010009). All participants provided written informed consent after receiving comprehensive information about the study.

### 2.2 Maternal Pre‐Pregnancy BMI and Classification

At recruitment, each participant completed a structured questionnaire that included items on height and pre‐pregnancy weight. Pre‐pregnancy body mass index (BMI) was calculated as weight in kilograms divided by height in meters squared (kg/m^2^). Based on Chinese adult standards, pre‐pregnancy overweight and obesity were defined as BMI ≥ 24.0 kg/m^2^ and ≥ 28.0 kg/m^2^, respectively. Given the generally low prevalence of obesity among highly educated women in economically developed urban areas of China [16], and the specific obesity rate of 2.1% (56/2639) in our study population, we combined overweight and obesity into a single category for subsequent analyses.

### 2.3 Maternal OGTT During Pregnancy and Modeling of OGTT Glucose Response Patterns

The 75‐g OGTT performed 24–28 weeks of gestation served as a primary method for assessing maternal glucose metabolism and screening for GDM [12]. Participants were instructed to fast for 8–10 h before the test while maintaining their usual diet (containing ≥ 150 g carbohydrates daily) for the preceding 3 days. On the test day, subjects remained seated and refrained from smoking while consuming 300 mL of a solution containing 75‐g anhydrous glucose within 5 min.

Venous blood samples were collected by trained nurses at fasting, 1‐ and 2‐h intervals following ingestion, with timing commencing upon initiation of the glucose solution consumption. The OGTT data used in this study were obtained from routine antenatal clinical care. During the study period, venous glucose concentrations were routinely measured at fasting, 1 and 2 h after glucose loading, while additional post‐load glucose measurements at 30 and 90 min, as well as insulin and C‐peptide concentrations, were not routinely collected. Therefore, the present analysis was based on the available three‐point OGTT glucose measurements only. Blood glucose levels were measured at each time point. All fasting blood samples were obtained before 9:00 a.m. to prevent prolonged fasting from influencing the results. GDM was diagnosed according to International Association of Diabetes and Pregnancy Study Groups (IADPSG) criteria [12], with cutoff values of 5.1 mmol/L (fasting), 10.0 mmol/L (1‐h), and 8.5 mmol/L (2‐h). A diagnosis was established when any value met or exceeded these thresholds. No pharmacological interventions or insulin therapy were administered to GDM patients in this cohort.

OGTT glucose response patterns were identified using group‐based trajectory modeling (GBTM) based on fasting, 1‐ and 2‐h glucose measurements from the standard 75‐g OGTT. GBTM is a finite mixture modeling approach that classifies individuals into latent subgroups with similar longitudinal response profiles [17]. Because only three OGTT time points were available in routine clinical practice, quadratic trajectories represented the highest‐order polynomial shape considered in the modeling process.

**FIGURE 2:**
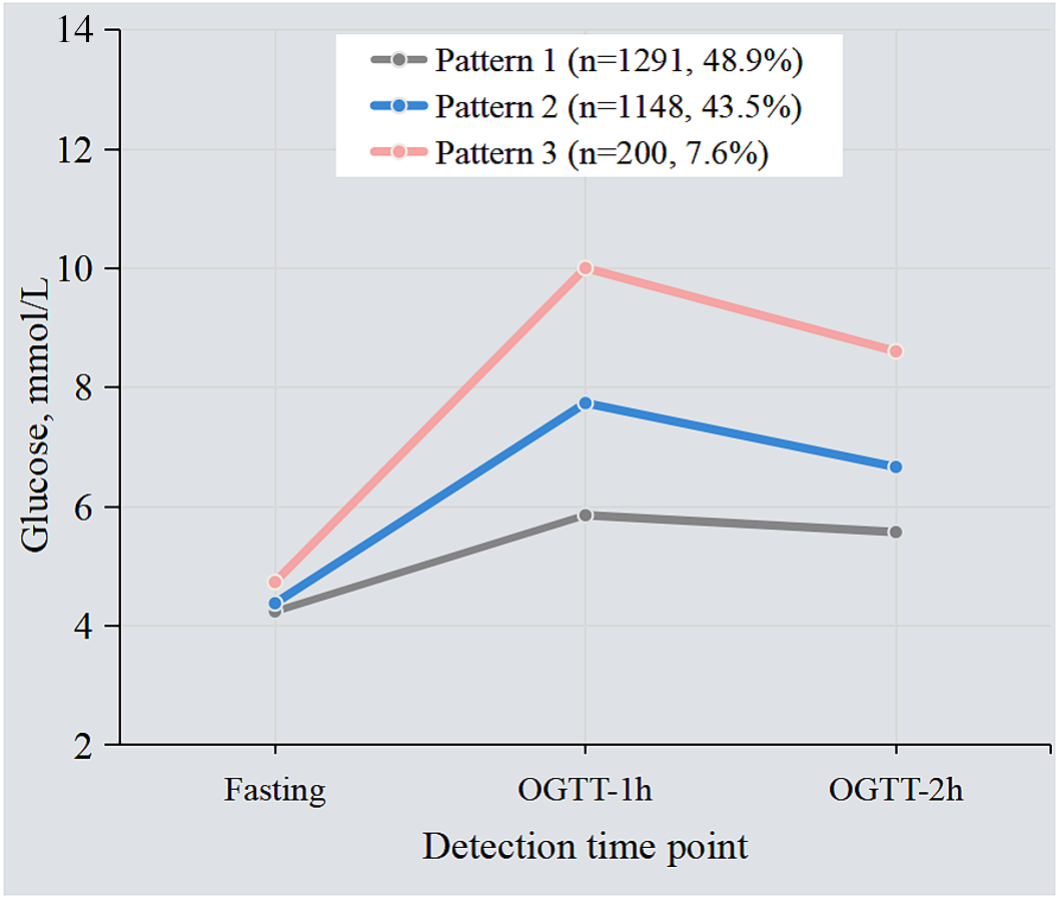
Three distinct OGTT response patterns identified by GBTM in pregnant women.

Candidate models with two, three, and four subgroups were fitted and compared. The optimal model was selected according to a combination of statistical and clinical criteria [17], including the Bayesian information criterion (BIC), 2ΔBIC between adjacent models, average posterior probability (AvePP) for each subgroup, subgroup size, and clinical interpretability of the response profiles. An AvePP greater than 0.70 was considered to indicate acceptable classification quality, and each subgroup was required to include more than 5% of the study population to avoid unstable or overfitted classifications. Three distinct OGTT response patterns were ultimately identified and classified as mild (pattern 1), moderate (pattern 2), or the most pronounced (pattern 3) based on fluctuation magnitude. These patterns were illustrated in Figure 2 and used in subsequent analyses; their distribution by maternal pre‐pregnancy BMI category is shown in Table S1, and detailed model‐fitting statistics are presented in Table S2.

### 2.4 Offspring Neurodevelopment Assessment at 2 Years of Age

Neurodevelopment was assessed using the BSID at the prespecified 2‐year follow‐up visit. Although widely applicable from 2 to 30 months of age [18], the 2‐year time point was selected in the cohort protocol to standardize assessment timing and improve comparability across participants. This age represents an important developmental window for evaluating early mental and psychomotor functions. This scale assesses nervous system development and functional maturity through three components: a mental scale (163 items), a psychomotor scale (81 items), and a behavioral record (24 items). The mental scale evaluates domains including perception, memory, learning, problem‐solving, vocalization, early language communication, and preliminary abstract thinking. The psychomotor scale measures gross motor abilities (such as sitting, standing, walking, and stair climbing) and fine motor skills involving hand and finger movements. The behavioral record documents the child's emotional responses and cooperation during assessment; these observations are not incorporated into the quantitative scores.

Administration of the mental and psychomotor scales yielded raw scores, which were converted to the Mental Development Index (MDI) and Psychomotor Development Index (PDI), respectively. Scores were derived through a standardized process: raw scores based on passed items were first converted to crude developmental indices using conversion tables, then transformed into standardized MDI and PDI scores normalized to a mean of 100 and a standard deviation (SD) of 15 within age‐specific groups. Both indices employ a seven‐tier classification system: < 70 (developmental delay), 70–79 (borderline), 80–89 (below average), 90–109 (average), 110–119 (above average), 120–129 (superior), and ≥ 130 (exceptional) [19]. For this study, suboptimal mental and psychomotor development were defined as MDI and PDI scores < 90, respectively. This cutoff was chosen to identify children whose scores fell below the average range of the BSID classification system, including below‐average, borderline, and delayed performance. Therefore, this definition was intended to capture suboptimal neurodevelopmental performance at the population level rather than to establish a clinical diagnosis of developmental delay.

### 2.5 Covariates

Data on covariates were obtained from structured questionnaires completed at enrollment and from electronic medical records at delivery. These included demographic characteristics, socioeconomic status, lifestyle factors, and medical history. Gestational weight gain was calculated as the difference between self‐reported pre‐pregnancy weight and measured weight at delivery, and was categorized as insufficient, appropriate, or excessive according to the Institute of Medicine (IOM) guidelines. The IOM criteria were used because the participating women delivered between May 2014 and May 2019, during which no domestic Chinese GWG classification standard was widely adopted in routine clinical practice. Therefore, the IOM criteria were consistent with the clinical practice context during the study period and allowed comparability with previous studies.

Based on previous literature [2, 11, 20], the following covariates were selected for adjustment in the analytical models: maternal age, educational level (high school or below/college degree or above), passive smoking during pregnancy (yes/no), parity (primipara/multipara), gestational weight gain (insufficient/appropriate/excessive), delivery mode (vaginal delivery/cesarean section), gestational age at birth, and child sex (boy/girl).

### 2.6 Statistical Analysis

Normally distributed continuous variables were expressed as mean ± standard deviation (SD) and compared using Student's *t*‐test, whereas categorical variables were summarized as frequencies (percentages) and compared using Chi‐square tests. The associations of study variables with neurodevelopmental indices (MDI and PDI) were assessed using generalized linear models, and associations with suboptimal neurodevelopment (dichotomized outcomes) were evaluated using logistic regression models.

Beta coefficient (*β*) with 95% confidence intervals (CIs) were estimated for developmental indices, and odds ratios (ORs) with 95% CIs were calculated for binary outcomes. All models were adjusted for maternal age, educational level, passive smoking during pregnancy, parity, gestational weight gain, and infant sex. Additionally, sex‐stratified analyses (boys versus girls) were performed.

We subsequently examined additive interactions between pre‐pregnancy BMI and OGTT response patterns in relation to neurodevelopmental outcomes. Participants were categorized into four mutually exclusive groups based on maternal pre‐pregnancy BMI (overweight/obesity versus not) and OGTT response pattern (pattern 3 versus not pattern 3): Group 1 (reference): non‐overweight/obesity and not pattern 3; Group 2: non‐overweight/obesity and pattern 3; Group 3: overweight/obesity and not pattern 3; Group 4: overweight/obesity and pattern 3.

Additive interactions for suboptimal neurodevelopment were quantified using the relative excess risk due to interaction (RERI) and the attributable proportion due to interaction (AP), calculated from logistic regression models [21, 22]. RERI estimates the extent to which the combined effect of two factors exceeds the sum of their individual effects, while AP represents the proportion of total risk attributable to their interaction. Positive additive interaction is indicated by RERI > 0 and AP > 0, whereas negative interaction is indicated by RERI < 0 and AP < 0. Statistical significance of additive interaction was determined when the 95% CI for either RERI or AP excluded zero.

All analyses were performed using SAS, version 9.4 (SAS Institute Inc) and R, version 4.1.1 (R Foundation for Statistical Computing). GBTM was implemented using the *PROC TRAJ* macro in SAS, and additive interaction measures were computed using the *epiR* package in R. To account for multiple comparisons in the independent‐effect analyses (Tables 2 and S3), Benjamini–Hochberg false discovery rate (FDR) correction was applied. Corrections were performed separately by outcome and population group, with the three exposure comparisons (pre‐pregnancy overweight/obesity, OGTT Pattern 2, and OGTT Pattern 3) adjusted together. Both nominal and FDR‐adjusted *p* values were reported. Two‐sided *p* values < 0.05 were considered statistically significant.

## 3 Results

### 3.1 General Characteristics of the Study Population

Table 1 presents the general characteristics of the 2639 mother‐infant pairs. The mean maternal age was 29.04 ± 3.57 years, with 13.91% classified as having pre‐pregnancy overweight/obesity and 8.98% diagnosed with GDM. The mean gestational age at OGTT was 25.73 ± 1.24 weeks when calculated using exact gestational age in weeks and days. Based on completed gestational weeks, the median gestational age was 25 weeks, with an interquartile range of 24–26 weeks and a range of 24–28 weeks. The numbers of participants undergoing OGTT at 24, 25, 26, 27, and 28 completed gestational weeks were 884, 714, 506, 374, and 161, respectively. Thus, all participants underwent OGTT within the standard 24–28‐week screening window. Mean OGTT glucose values during pregnancy were 4.34, 7.00, and 6.29 mmol/L at fasting, 1‐h, and 2‐h time points, respectively, with significant sex differences observed only at the 1‐h measurement. At the scheduled 2‐year assessment, girls had higher age‐standardized MDI scores than boys, whereas no significant sex difference was observed for PDI scores. The prevalence of both suboptimal mental and psychomotor development was lower in girls compared to boys.

**TABLE 1:** General characteristics of the study population.

Characteristics	Mean ± SD/numbers (%)			*p*
	Total (*n* = 2639)	Boys (*n* = 1368)	Girls (*n* = 1271)	
*Mother's characteristics*				
Maternal age (years)	29.04 ± 3.57	29.04 ± 3.54	29.04 ± 3.59	0.960
Education level				
College degree or above	2156 (81.70)	1113 (81.36)	1043 (82.06)	0.641
High school or below	483 (18.30)	255 (18.64)	228 (17.94)	
Passive smoking during pregnancy				
No	2491 (94.39)	1285 (93.93)	1206 (94.89)	0.288
Yes	148 (5.61)	83 (6.07)	65 (5.11)	
Parity				
Primipara	2171 (82.27)	1105 (80.77)	1066 (83.87)	0.037
Multipara	468 (17.73)	263 (19.23)	205 (16.13)	
Pre‐pregnancy BMI (kg/m^2^)				
< 24.0	2272 (86.09)	1178 (86.11)	1094 (86.07)	0.978
≥ 24.0	367 (13.91)	190 (13.89)	177 (13.93)	
GWG				
Insufficient	102 (3.87)	55 (4.02)	47 (3.70)	0.826
Appropriate	946 (35.85)	495 (36.18)	451 (35.48)	
Excessive	1591 (60.29)	818 (59.80)	773 (60.82)	
Gestational age at OGTT (weeks)^a^	25.73 ± 1.24	25.73 ± 1.24	25.73 ± 1.25	0.984
Glucose value of OGTT				
Fasting (mmol/L)	4.34 ± 0.43	4.34 ± 0.44	4.34 ± 0.42	0.776
1 h (mmol/L)	7.00 ± 1.54	7.07 ± 1.58	6.93 ± 1.50	0.017
2 h (mmol/L)	6.29 ± 1.24	6.31 ± 1.24	6.26 ± 1.24	0.293
OGTT response patterns				
Pattern 1	1291 (48.92)	641 (46.86)	650 (51.14)	0.084
Pattern 2	1148 (43.50)	617 (45.10)	531 (41.78)	
Pattern 3	200 (7.58)	110 (8.04)	90 (7.08)	
GDM				
No	2402 (91.02)	1238 (90.50)	1164 (91.58)	0.330
Yes	237 (8.98)	130 (9.50)	107 (8.42)	
Delivery mode				
Vaginal delivery	1327 (50.28)	655 (47.88)	672 (52.87)	0.010
Cesarean section	1312 (49.72)	713 (52.12)	599 (47.13)	
*Children's characteristics*				
Gestational age at birth (weeks)	39.47 ± 0.95	39.41 ± 0.94	39.53 ± 0.94	< 0.001
MDI of BSID at age 2 (scores)	108.12 ± 22.45	104.59 ± 23.92	111.87 ± 20.12	< 0.001
Suboptimal mental development				
No	2087 (80.95)	1003 (75.47)	1084 (86.79)	< 0.001
Yes	491 (19.05)	326 (24.53)	165 (13.21)	
PDI of BSID at age 2 (scores)	107.52 ± 18.40	107.18 ± 19.02	107.88 ± 17.71	0.333
Suboptimal psychomotor development				
No	2150 (84.35)	1087 (82.72)	1063 (86.07)	0.020
Yes	399 (15.65)	227 (17.28)	172 (13.93)	

Abbreviations: BMI, body mass index; BSID, Bayley Scale of Infant Development; GDM, gestational diabetes; GWG, gestational weight gain; MDI, mental development index; OGTT, oral glucose tolerance test; PDI, psychomotor development index.

^a^ Gestational age at OGTT was calculated using exact gestational age expressed continuously in weeks and days. Based on completed gestational weeks, the median gestational age at OGTT was 25 weeks, with an interquartile range of 24–26 weeks and a range of 24–28 weeks. The numbers of participants undergoing OGTT at 24, 25, 26, 27, and 28 completed gestational weeks were 884, 714, 506, 374, and 161, respectively.

### 3.2 OGTT Response Patterns Identified by GBTM

Three distinct OGTT response patterns were identified through GBTM and are illustrated in Figure 2. The model‐fitting statistics for the candidate two‐, three‐, and four‐group models are shown in Table S2. The three‐group model showed good classification quality, with AvePP values of 0.90, 0.88, and 0.91, and all subgroups included more than 5% of participants. Although the four‐group model showed a further improvement in fit indices, it generated a very small subgroup comprising only 2.8% of the study population, suggesting potential overfitting and limited stability for subsequent stratified analyses. The two‐group model was stable but did not adequately distinguish the heterogeneity of post‐load glucose responses. Therefore, the three‐group model was selected as the final model based on the balance between statistical fit, classification quality, subgroup size, and clinical interpretability. Pattern 1, characterized by normal glucose tolerance, represented the largest subgroup (48.92%). Pattern 2, comprising 43.50% of participants, exhibited elevated glucose levels at both 1‐ and 2‐h time points following glucose challenge. Pattern 3, accounting for 7.6% of participants, showed the most pronounced post‐load glycemic response and was therefore used to represent the highest OGTT response pattern in subsequent joint‐exposure analyses.

### 3.3 Associations of Pre‐Pregnancy BMI and OGTT Measures With Offspring Neurodevelopment

As summarized in Table 2, maternal pre‐pregnancy overweight/obesity showed nominal associations with lower MDI scores in the overall population (*β* = −2.60, 95% CI: −5.07 to −0.13; nominal *p* = 0.039; FDR‐adjusted *p* = 0.117) and in girls (*β* = −3.47, 95% CI: −6.67 to −0.27; nominal *p* = 0.034; FDR‐adjusted *p* = 0.102). However, these associations did not remain statistically significant after FDR correction. No corresponding association was observed in boys. OGTT response patterns alone were not associated with MDI or PDI in the overall population or in sex‐stratified analyses after adjustment and FDR correction. Similarly, neither maternal pre‐pregnancy overweight/obesity nor OGTT response patterns were significantly associated with suboptimal mental or psychomotor development after FDR correction (Table S3). Therefore, the independent‐effect findings should be interpreted cautiously.

**TABLE 2:** Associations of maternal pre‐pregnancy BMI and OGTT response patterns with neurodevelopmental indices in offspring at 2 years of age.

Category	All			Boys			Girls		
	*β* (95% CI)	*p*	FDR‐adjusted P	*β* (95% CI)	*p*	FDR‐adjusted P	*β* (95% CI)	*p*	FDR‐adjusted P
*MDI of BSID*									
Pre‐pregnancy BMI (kg/m^2^)									
< 24.0	Reference			Reference			Reference		
≥ 24.0	**−2.60 (−5.07, −0.13)**	**0.039**	0.117	−1.86 (−5.61, 1.88)	0.329	0.987	**−3.47 (−6.67, −0.27)**	**0.034**	0.102
Shape of OGTT response curves									
Pattern 1	Reference			Reference			Reference		
Pattern 2	0.62 (−1.18, 2.41)	0.502	0.753	0.01 (−2.72, 2.72)	0.999	0.999	1.28 (−1.05, 3.61)	0.282	0.423
Pattern 3	0.07 (−3.45, 3.32)	0.997	0.997	0.63 (−4.39, 5.64)	0.806	0.999	−0.96 (−5.44, 3.53)	0.676	0.676
*PDI of BSID*									
Pre‐pregnancy BMI (kg/m^2^)									
< 24.0	Reference			Reference			Reference		
≥ 24.0	0.29 (−1.78, 2.36)	0.782	0.782	0.77 (−2.21, 3.75)	0.614	0.976	−0.18 (−3.04, 2.69)	0.903	0.903
Shape of OGTT response curves									
Pattern 1	Reference			Reference			Reference		
Pattern 2	0.68 (−0.82, 2.18)	0.371	0.782	−0.03 (−2.20, 2.13)	0.976	0.976	1.49 (−0.58, 3.55)	0.159	0.477
Pattern 3	0.43 (−2.40, 3.26)	0.765	0.782	0.22 (−3.75, 4.19)	0.914	0.976	0.63 (−3.40, 4.66)	0.760	0.903

*Note:* FDR‐adjusted *p* values were calculated using the Benjamini–Hochberg procedure within each analytical family defined by the same study population and the same neurodevelopmental outcome. Within each family, the three exposure comparisons were corrected together: pre‐pregnancy overweight/obesity versus non‐overweight/obesity, Pattern 2 versus Pattern 1, and Pattern 3 versus Pattern 1. The models were adjusted for maternal age, educational level, passive smoking during pregnancy, parity, gestational age at birth, and infant sex. In the results stratified by infant sex, the model adjusted for the same covariates above except for infant sex. Bold values denote statistically significant differences.

Abbreviations: BMI, body mass index; BSID, Bayley Scale of Infant Development; MDI, mental development index; OGTT, Oral glucose tolerance test; PDI, psychomotor development index.

### 3.4 Combined Associations of Pre‐Pregnancy Overweight/Obesity and OGTT Response Patterns With Offspring Neurodevelopment

**FIGURE 3:**
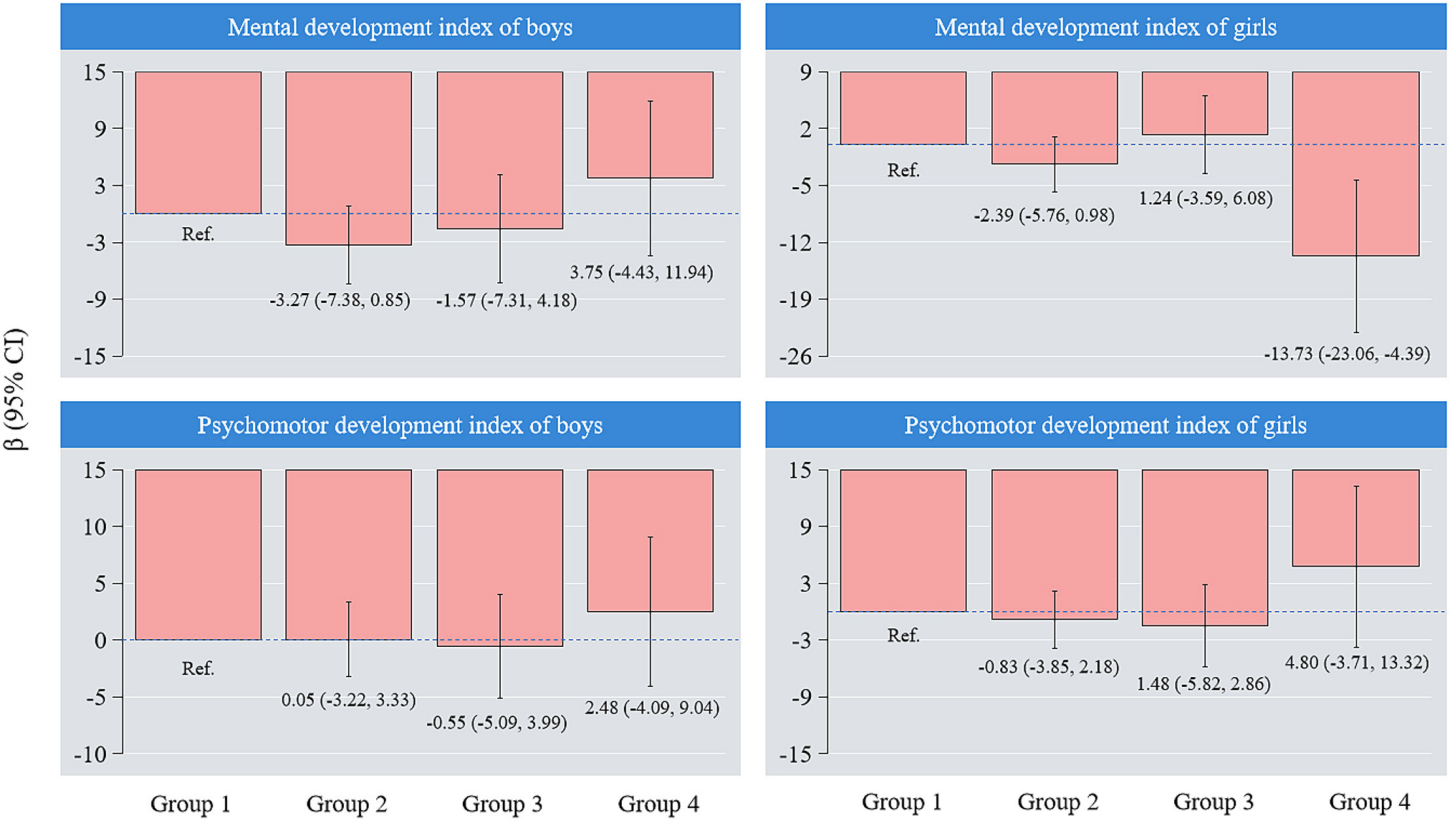
Joint associations of maternal pre‐pregnancy BMI and OGTT response patterns with neurodevelopmental indices in offspring at 2 years of age. BMI, body mass index; OGTT, oral glucose tolerance test. The models were adjusted for maternal age, educational level, passive smoking during pregnancy, parity, and gestational age at birth. Group 1 (reference): Non‐overweight/obesity and not pattern 3; Group 2: Non‐overweight/obesity and pattern 3; Group 3: Overweight/obesity and not pattern 3; Group 4: Overweight/obesity and pattern 3.

**FIGURE 4:**
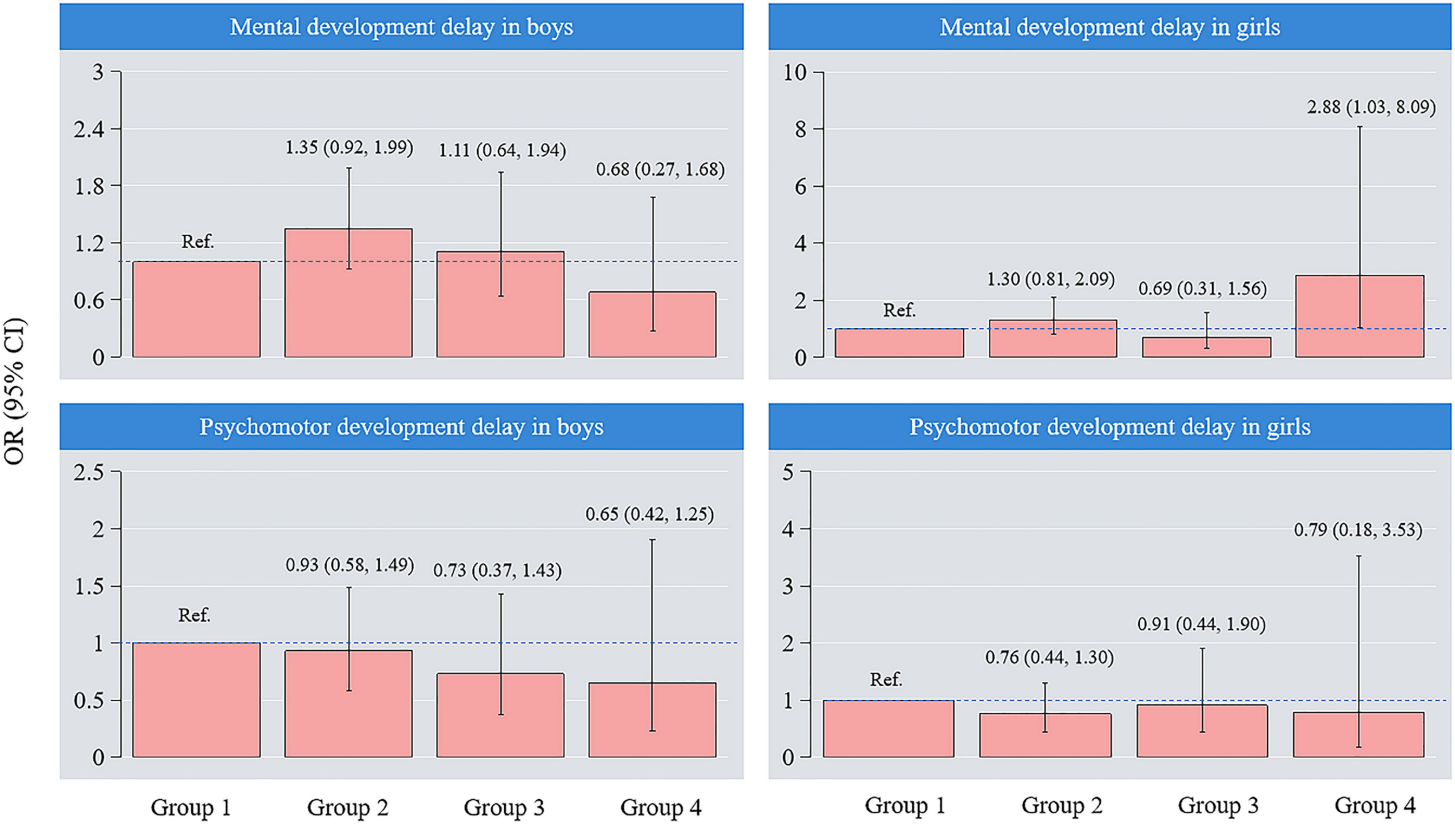
Joint associations of maternal pre‐pregnancy BMI and OGTT response patterns with suboptimal neurodevelopment in offspring at 2 years of age. BMI, body mass index; OGTT, oral glucose tolerance test. The models were adjusted for maternal age, educational level, passive smoking during pregnancy, parity, and gestational age at birth. Group 1 (reference): Non‐overweight/obesity and not pattern 3; Group 2: Non‐overweight/obesity and pattern 3; Group 3: Overweight/obesity and not pattern 3; Group 4: Overweight/obesity and pattern 3.

The combined associations of maternal pre‐pregnancy BMI and OGTT response patterns with offspring neurodevelopmental outcomes are presented in Figures 3 and 4. Compared with the reference group, girls exposed to both maternal pre‐pregnancy overweight/obesity and Pattern 3 showed a markedly lower MDI score (*β* = −13.73, 95% CI: −23.06 to −4.39, *p* = 0.006) and a higher risk of suboptimal mental development (OR = 2.88, 95% CI: 1.03 to 8.09, *p* = 0.038). In boys, the corresponding joint exposure was not statistically significantly associated with suboptimal mental development (OR = 0.68, 95% CI: 0.27 to 1.68, *p* = 0.346), and the association with continuous MDI was also not statistically significant (*β* = 3.75, 95% CI: −4.43 to 11.94, *p* = 0.350).

As shown in Table 3, positive additive interaction indices for suboptimal mental development were observed in both boys (RERI = 0.74, 95% CI: 0.23 to 1.26; AP = 0.46, 95% CI: 0.28 to 0.64) and girls (RERI = 0.76, 95% CI: 0.03 to 1.49; AP = 0.42, 95% CI: 0.17 to 0.67). However, because the male‐specific additive interaction was not supported by a statistically significant joint‐effect estimate or by consistent findings for continuous MDI, it was interpreted cautiously as an exploratory signal. The evidence for an adverse combined effect was more consistent among girls.

**TABLE 3:** Additive interaction between maternal pre‐pregnancy BMI and OGTT response patterns on suboptimal neurodevelopment in offspring at 2 years of age.

Outcomes	Joint‐effect OR (95% CI)	RERI (95% CI)	AP (95% CI)
Suboptimal mental development			
Boys	0.68 (0.27, 1.68)	**0.74 (0.23, 1.26)**	**0.46 (0.28, 0.64)**
Girls	**2.88 (1.03, 8.09)**	**0.76 (0.03, 1.49)**	**0.42 (0.17, 0.67)**
Suboptimal psychomotor development			
Boys	0.65 (0.42, 1.25)	0.29 (−0.16, 0.75)	0.24 (−0.05, 0.54)
Girls	0.79 (0.18, 3.53)	0.01 (−0.48, 0.48)	0.01 (−0.46, 0.47)

*Note:* The bold values indicate statistical significance, and the additive interaction indices should be interpreted together with the joint‐effect estimate for the combined exposure group. Logistic models were adjusted for maternal age, educational level, passive smoking during pregnancy, parity, and gestational age at birth. Both pre‐pregnancy BMI (overweight/obesity versus non‐overweight/obesity) and shape of OGTT response curve pattern (pattern 3 versus not pattern 3) were modeled as binary variables.

Abbreviations: AP, attributable proportion due to interaction; BMI, body mass index; OGTT, oral glucose tolerance test; RERI relative excess risk due to interaction.

## 4 Discussion

This prospective cohort study utilizing healthcare and follow‐up data from 2639 mother–child pairs investigated the independent and combined associations of maternal pre‐pregnancy BMI and OGTT response patterns with neurodevelopment in 2‐year‐old children. In the independent‐effect analyses, maternal pre‐pregnancy overweight/obesity showed nominal associations with lower MDI scores in the overall population and in girls, but these associations were not significant after FDR correction. OGTT response patterns alone were not associated with neurodevelopmental outcomes after FDR correction. The main finding was from the joint‐exposure analysis, where girls with both maternal pre‐pregnancy overweight/obesity and the most pronounced OGTT response pattern had lower MDI scores and a higher risk of suboptimal mental development. To our knowledge, this is among the first studies in a Chinese prospective birth cohort to report synergistic adverse effects between maternal pre‐pregnancy overweight/obesity and dysglycemia during pregnancy on offspring neurodevelopment, as evidenced by a 13.73‐point decrease in MDI and a 2.88‐fold increased risk of suboptimal mental development among 2‐year‐old girls exposed to both risk factors.

Overweight and obesity in women of reproductive age represent a major global public health concern. Accumulating evidence confirms that elevated maternal pre‐pregnancy BMI increases the risk of neuropsychological abnormalities in offspring. A Chinese cohort study from Tianjin involving 2253 mother–child pairs demonstrated that high pre‐pregnancy BMI (≥ 25 kg/m^2^) significantly impaired developmental quotient in 2‐year‐old children [3]. Similarly, the U.S. Collaborative Perinatal Project reported an inverted U‐shaped relationship between maternal pre‐pregnancy BMI and child intelligence quotient, with optimal scores observed at approximately 20 kg/m^2^ [2]. Danish research (*n* = 4094) indicated that compared to offspring of mothers with normal pre‐pregnancy weight (18.5 ≤ BMI < 25.0), children of overweight (25 ≤ BMI < 30.0) and obesity (BMI ≥ 30.0) mothers had 32% and 78% higher risks of behavioral problems at age 5, respectively [23]. A U.S. case–control study of 2036 children assessed at 2–5 years of age found that maternal pre‐pregnancy obesity (BMI ≥ 30.0) was associated with adjusted ORs of 1.37 (95% CI: 0.98–1.92) for autism spectrum disorders and 1.48 (95% CI: 1.08–2.02) for other developmental delays [24]. Although these studies employed different BMI cut‐offs and assessed varied neuropsychological outcomes compared to our study (which used a BMI cut‐off of 24.0 kg/m^2^ for the Chinese population), their findings collectively align with our results.

Maternal overweight/obesity adversely affects glucose metabolism during pregnancy. Overweight, obesity, and severely obese pregnant women face 2.14‐fold, 3.56‐fold, and 8.56‐fold increased risks of developing GDM, respectively, compared to normal‐weight women [25]. Previous studies have consistently documented the adverse effects of GDM on offspring neurodevelopment. Importantly, even subdiagnostic hyperglycemia during pregnancy can alter fetal growth trajectories [26] and increase the risk of adverse birth outcomes, including macrosomia, preterm birth, and small‐for‐gestational‐age infants [27].

In our study, OGTT response patterns alone showed no independent association with neurodevelopmental scores or suboptimal neurodevelopment risk. However, when considered jointly with pre‐pregnancy BMI, significant associations emerged. Among 2‐year‐old girls exposed to both maternal pre‐pregnancy overweight/obesity and adverse glucose metabolism, 42% (95% CI: 17%–67%) of the risk for suboptimal mental development was attributable to the interaction between these factors. Previous research has demonstrated that maternal hyperglycemia below the diagnostic threshold for GDM still exhibits a strong, continuous association with increased risks of adverse birth outcomes [28]. The most pronounced OGTT response curve pattern likely reflects impaired pancreatic *β*‐cell function [14, 15], reduced insulin sensitivity [13], and heightened insulin resistance [29].

Several hypothesized mechanisms may explain the association between pre‐pregnancy overweight/obesity, gestational glucose dysregulation, and adverse neurodevelopment. Pregnant women with overweight/obesity may exhibit elevated circulating proinflammatory cytokines that promote placental and intrauterine inflammation, potentially increasing fetal neuronal injury risk [30]. Maternal hyperglycemia not only exacerbates intrauterine inflammation [31] but also induces oxidative stress [32], which may disrupt epigenetic programming and impair fetal neurodevelopment [33]. Animal studies suggest that transplacental glucose transfer leads to fetal hyperinsulinemia and insulin resistance, potentially compromising brain regions crucial for cognition that are rich in insulin receptors [34]. Additionally, maternal hyperglycemia may impair placental uptake of long‐chain polyunsaturated fatty acids [35, 36] or downregulate leptin receptors during critical periods of fetal brain development [37, 38], thereby contributing to cognitive deficits in offspring.

Although positive additive interaction indices were also observed in boys, these findings should be interpreted cautiously. The lack of significant associations for the joint exposure and continuous MDI outcome suggests that the male‐specific RERI and AP estimates may represent exploratory signals rather than robust evidence of an adverse combined effect, possibly due to limited subgroup size or chance variation.

In contrast, findings in girls were more consistent, with joint exposure associated with both lower MDI scores and higher risk of suboptimal mental development. Therefore, our main interpretation focused on the more consistent adverse combined effect observed among girls, while the potential sex‐specific effects warrant further investigation. Sex‐specific epigenetic modifications may partly explain this finding. Females may exhibit greater sensitivity to leptin signaling, potentially amplifying the detrimental effects of abnormal intrauterine nutrition on neurodevelopment [39]. Estrogen's neuroprotective role in females represents another potential mechanism [40, 41]. Maternal metabolic disturbances such as hyperinsulinemia might interfere with estrogen receptor signaling, diminishing resistance to oxidative stress and inflammation. The observation that girls had higher MDI scores than boys at the 2‐year assessment [42] should be interpreted cautiously. This finding reflects a descriptive difference in age‐standardized developmental performance at a fixed follow‐up visit rather than direct evidence that girls had a faster neurodevelopmental pace. The nominal association between maternal pre‐pregnancy overweight/obesity and lower MDI scores in girls was estimated within the female subgroup and therefore was not simply driven by the overall sex difference in MDI scores. However, because this independent association did not remain statistically significant after FDR correction, it should be regarded as exploratory. Larger cohorts with repeated neurodevelopmental assessments are needed to determine whether maternal metabolic exposures are associated with sex‐specific developmental trajectories over time.

This study benefits from its prospective cohort design and standardized neurodevelopmental assessments. However, several limitations warrant consideration. First, OGTT was performed only at 24–28 weeks' gestation, precluding evaluation of glucose metabolism during earlier pregnancy stages when fetal brain development initiates. Second, the OGTT protocol included only three glucose measurements (fasting, 1‐ and 2‐h post‐load values), which limited our ability to characterize complex glucose curve phenotypes, such as monophasic or biphasic trajectories [15, 43, 44]. Therefore, the identified OGTT patterns should be interpreted as routine clinical glucose response profiles rather than comprehensive dynamic trajectories. Future studies incorporating additional OGTT sampling time points (e.g., 30, 60, 90, and 120 min) and insulin or C‐peptide measurements are needed to better characterize glucose dynamics and their associations with early‐life neurodevelopment. Third, we used MDI or PDI scores < 90 to indicate suboptimal development, covering below‐average, borderline, or delayed performance. This threshold increases sensitivity for early vulnerability but lowers specificity for clinically significant impairment, potentially overestimating clinically meaningful delay if viewed diagnostically. However, the goal was population‐level evaluation, not diagnosis. Future studies require stricter clinical cutoffs, formal diagnostics, and longer follow‐up to clarify persistence and clinical significance. Fourthly, GWG was categorized according to the IOM guidelines rather than Chinese‐specific criteria. Although this choice reflected clinical practice during the study period, the IOM recommendations were developed primarily based on Western populations and may not fully represent GWG patterns among Chinese women. This may have caused some GWG misclassification, partly explaining the high proportion of excessive GWG in this cohort and limiting generalizability to populations using other criteria. Because GWG was included as an adjustment covariate rather than the primary exposure, this issue mainly affects the external validity of the findings. In addition, although we adjusted for multiple potential confounders, residual confounding cannot be completely excluded because of the observational design. Finally, this was a hospital‐based cohort from a single region and predominantly included a single ethnic population, which may further limit the generalizability of the findings to other populations.

## 5 Conclusions

Our findings suggest that maternal pre‐pregnancy overweight/obesity and OGTT response patterns alone had limited independent associations with offspring neurodevelopment after FDR correction. However, combined exposure to pre‐pregnancy overweight/obesity and the most pronounced OGTT response pattern may be associated with poorer mental developmental performance in offspring. Although positive additive interaction indices were also observed in boys, the evidence for an adverse combined effect was more consistent in girls, as reflected by both lower continuous MDI scores and a higher risk of suboptimal mental development. These findings highlight the potential importance of pre‐pregnancy weight management and glycemic monitoring during pregnancy, while further studies are needed to validate the observed sex‐specific patterns.

## Author Contributions

K.C., X.F. and Y.W. made substantial contributions to the conception and design of the study, data analysis, drafting of the manuscript and served as the equally contributing first authors of the manuscript. C.X. and A.Z. made substantial contributions to intellectual direction and revision of the drafting of the manuscript. Z.C., L.X., X.Q., M.H., and X.M. made contributions to data collection and revision of the drafting of the manuscript. All authors read and approved the final manuscript.

## Funding

This study was supported by the Knowledge Innovation Specialized Project of Wuhan Science and Technology Bureau (2023020201010198), National Natural Science Foundation of China (81903331), and Hubei Provincial Science and Technology Plan Project for Clinical Research Center of Neurodevelopmental Disorders in Children (No. 2022DCC020).

## Ethics Statement

This study was approved by the Medical Ethics Committees of Wuhan Children's Hospital (Wuhan Maternal and Child Healthcare Hospital), Tongji Medical College, Huazhong University of Science and Technology (Approve Number: 2010009).

## Consent

All participants were informed and signed an informed consent form.

## Conflicts of Interest

The authors declare no conflicts of interest.

## Supporting information


**Table S1:** Distribution of OGTT response patterns by maternal pre‐pregnancy BMI category.
**Table S2:** Model fitting statistics for different numbers of OGTT response patterns identified by GBTM.
**Table S3:** Associations of maternal pre‐pregnancy BMI and OGTT response patterns with suboptimal neurodevelopment in offspring at 2 years of age.

## Data Availability

The datasets generated and/or analyzed during this study are available from the corresponding author upon reasonable request.
