# Supplementary material for: Sex Differences in Combined Effects of Maternal Pre‐Pregnancy BMI and OGTT Response Patterns on Early Neurodevelopment in Offspring: A Cohort Study
Source: J Diabetes. 2026 Aug 11;18(8):e70261. doi: 10.1111/1753-0407.70261 (PMC13459868; doi:10.1111/1753-0407.70261)
Supplement: Supplementary file 1 [file JDB-18-e70261-s001.docx]

**Title:** Sex Differences in Combined Effects of Maternal Pre-pregnancy BMI and OGTT Response Patterns on Early Neurodevelopment in Offspring: A Cohort Study

**Supplementary materials for review**

**Table S1.** Distribution of OGTT response patterns by maternal pre-pregnancy BMI category.

**Table S2.** Model fitting statistics for different numbers of OGTT response patterns identified by GBTM.

**Table S3.** Associations of maternal pre-pregnancy BMI and OGTT response patterns with suboptimal neurodevelopment in offspring at 2 years of age.

**Table S1. Distribution of OGTT response patterns by maternal pre-pregnancy BMI category.**

| **Variables** | **Total**  **(n = 2639, 100%)** | **Pattern 1**  **(n = 1291, 48.9%)** | **Pattern 2**  **(n = 1148, 43.5%)** | **Pattern 3**  **(n = 200, 7.6%)** | ***P*-value** |
| --- | --- | --- | --- | --- | --- |
| **Pre-pregnancy BMI** |  |  |  |  |  |
| < 24.0 kg/m^2^ | 2272 (100.0%) | 1172 (51.6%) | 953 (41.9%) | 147 (6.5%) | ＜0.001 |
| ≥ 24.0 kg/m^2^ | 367 (100.0%) | 119 (32.4%) | 195 (53.1%) | 53 (14.4%) |  |

**Table S2. Model fitting statistics for different numbers of OGTT response patterns identified by GBTM.**

| **Model fitting information** | **Numbers of subgroups for shape of OGTT response curve** | | |
| --- | --- | --- | --- |
|  | **2** | **3** | **4** |
| BIC | -11494.9 | **-11070.2** | -10895.0 |
| 2ΔBIC | - | **849.4** | 350.4 |
| Optimal fit morphology of each subgroup | 2/2 | **2/2/2** | 2/2/2/2 |
| AvePP of each subgroup | 0.96/0.90 | **0.90/0.88/0.91** | 0.86/0.87/0.85/0.90 |
| Number of participants in each subgroup | 1936/703 | **1291/1148/200** | 800/477/1289/73 |
| Proportion of each subgroup, % | 73.4/26.6 | **48.9/43.5/7.6** | 30.3/18.1/48.8/2.8 |

**Table S3. Associations of maternal pre-pregnancy BMI and OGTT response patterns with suboptimal neurodevelopment in offspring at 2 years of age.**

| **Category** | **All** | | |  | **Boys** | | |  | **Girls** | | |
| --- | --- | --- | --- | --- | --- | --- | --- | --- | --- | --- | --- |
|  | **OR (95% CI)** | **P** | **FDR-adjusted P** |  | **OR (95% CI)** | **P** | **FDR-adjusted P** |  | **OR (95% CI)** | **P** | **FDR-adjusted P** |
| **Suboptimal mental development** | |  |  |  |  |  |  |  |  |  |  |
| Pre-pregnancy BMI (kg/m^2^) | |  |  |  |  |  |  |  |  |  |  |
| < 24.0 | Reference |  |  |  | Reference |  |  |  | Reference |  |  |
| ≥ 24.0 | 1.27 (0.96, 1.68) | 0.088 | 0.264 |  | 1.18 (0.83, 1.69) | 0.357 | 0.832 |  | 1.44 (0.94, 2.23) | 0.098 | 0.294 |
| Shape of OGTT response curves | |  |  |  |  |  |  |  |  |  |  |
| Pattern 1 | Reference |  |  |  | Reference |  |  |  | Reference |  |  |
| Pattern 2 | 0.97 (0.79, 1.20) | 0.779 | 0.779 |  | 1.07 (0.82, 1.40) | 0.605 | 0.832 |  | 0.81 (0.57, 1.16) | 0.249 | 0.374 |
| Pattern 3 | 0.94 (0.63, 1.40) | 0.763 | 0.779 |  | 0.95 (0.57, 1.56) | 0.832 | 0.832 |  | 0.94 (0.49, 1.80) | 0.856 | 0.856 |
| **Suboptimal psychomotor development** | |  |  |  |  |  |  |  |  |  |  |
| Pre-pregnancy BMI (kg/m^2^) | |  |  |  |  |  |  |  |  |  |  |
| < 24.0 | Reference |  |  |  | Reference |  |  |  | Reference |  |  |
| ≥ 24.0 | 0.81 (0.58, 1.13) | 0.217 | 0.435 |  | 0.85 (0.55, 1.32) | 0.461 | 0.692 |  | 0.76 (0.46, 1.26) | 0.284 | 0.683 |
| Shape of OGTT response curves |  |  |  |  |  |  |  |  |  |  |  |
| Pattern 1 | Reference |  |  |  | Reference |  |  |  | Reference |  |  |
| Pattern 2 | 0.95 (0.76, 1.19) | 0.664 | 0.664 |  | 0.99 (0.73, 1.35) | 0.965 | 0.965 |  | 0.90 (0.64, 1.26) | 0.537 | 0.683 |
| Pattern 3 | 0.78 (0.50, 1.23) | 0.290 | 0.435 |  | 0.74 (0.40, 1.34) | 0.317 | 0.692 |  | 0.87 (0.44, 1.72) | 0.683 | 0.683 |

Note: FDR-adjusted P values were calculated using the Benjamini–Hochberg procedure within each analytical family defined by the same study population and the same neurodevelopmental outcome. Within each family, the three exposure comparisons were corrected together: pre-pregnancy overweight/obesity vs. non-overweight/obesity, Pattern 2 vs. Pattern 1, and Pattern 3 vs. Pattern 1.

BSID, Bayley Scale of Infant Development; MDI, mental development index; PDI, psychomotor development index; BMI, body mass index; OGTT, Oral glucose tolerance test. The models were adjusted for maternal age, educational level, passive smoking during pregnancy, parity, and gestational age at birth. In the results stratified by infant sex, the model adjusted for the same covariates above except for infant sex.
